# A young girl with an unusual cause of acute kidney injury: Answers

**DOI:** 10.1007/s00467-015-3171-x

**Published:** 2015-08-20

**Authors:** Mathijs Binkhorst, Kioa L. Wijnsma, Eric J. Steenbergen, Nicole C. A. J. van de Kar, Michiel F. Schreuder

**Affiliations:** 1 Department of Paediatrics, Amalia Children’s Hospital, Radboud University Medical Centre, P.O. Box 1901, 6500 HB Nijmegen, Netherlands; 2 Department of Paediatric Nephrology, Amalia Children’s Hospital, Radboud University Medical Centre, Nijmegen, Netherlands; 3 Department of Pathology, Radboud University Medical Centre, Nijmegen, Netherlands

**Keywords:** Acute interstitial nephritis, Haemorrhagic, Acute kidney injury, Hantavirus, Group A streptococcus, Children

Table 1 lists the differential diagnosis of acute interstitial nephritis (AIN).

## Answers

1. The parents of our patient denied the use of any medication. Blood and biopsy analysis did not reveal eosinophilia. Thus, drug-induced acute interstitial nephritis (AIN) was highly unlikely in this young girl. Neither the clinical presentation nor the radiographic, laboratory or histological findings supported a diagnosis of auto-immune disease. Tubulointerstitial nephritis and uveitis (TINU) syndrome was excluded since ophthalmic examination showed no signs of uveitis. Normal results for complement and immunoglobulin studies refuted other rare aetiologies. AIN was probably caused by an infectious agent in our patient, taking into account the high fever, high C-reactive protein level and elevated blood leukocytes. Several microorganisms were clinically and epidemiologically implausible and, therefore, not specifically studied. Others were tested and found to be negative. Because the clinical presentation evidently exemplified a case of scarlet fever and the blood culture was positive for *Streptococcus pyogenes*, AIN could be ascribed to streptococcal infection in our patient.
2. Haemorrhagic interstitial nephritis (HIN) can be caused by (hyper)acute humoral rejection, renal infarction, invasive procedures (e.g. needle biopsy) and various infections, among which are leptospirosis, rickettsiosis, hantavirus-associated haemorrhagic fever with renal syndrome (HFRS) and other haemorrhagic fever viruses (Marburg, Ebola and Flaviviridae) [1]. With the noteworthy exception of hantavirus, all of these infectious agents seemed unlikely in the present case. Hantavirus was first considered to be the culprit in our patient for the following reasons: (1) HFRS is a well-known and relatively frequent cause of acute haemorrhagic interstitial nephritis [2]; (2) all five phases of HFRS were discernible in our patient, with a period of polyuria prior to full recovery [1–5]; (3) anti-hantavirus immunoglobulin M (IgM) antibodies were initially (weakly) positive; (4) the young girl lives in a region of The Netherlands where hantavirus-infected rodents occur [4, 6], and direct contact with these animals is not a prerequisite for human infection since transmission of disease takes place via inhalation of aerosols contaminated by virus-infected rodent excreta [3, 6]. However, HFRS was eventually discarded as a diagnosis because certain clinical manifestations (e.g. thrombocytopenia) were absent and, more importantly, IgG for hantavirus remained negative [1, 3–5]. Whereas specific serum antibodies may incidentally be lacking in hantavirus-infected patients [7], an attempt to detect hantaviral antigen in the renal biopsy specimen was undertaken using reverse transcription PCR. Negative results were also obtained with this test. Streptococci are not listed among the causes of HIN. This may actually be the first report of acute haemorrhagic interstitial nephritis due to streptococcal infection.
3. There are three mechanisms by which streptococcal infection can conduce to acute kidney injury (AKI) [8]. First, acute post-streptococcal glomerulonephritis (APSGN) may result from scarlet fever or group A streptococcal skin infections. Inasmuch as the glomerular structure appeared to be unaffected in the renal biopsy specimen, hypertension was absent and serum complement levels were normal, APSGN was not the cause of AKI in this young girl. Second, fulminant streptococcal sepsis is commonly associated with ischaemic acute tubular necrosis (ATN) due to poor renal perfusion. Although this may have contributed to the deterioration of renal function in our patient, it was probably not the main cause, since tubular epithelial cell shedding was not found in the renal biopsy specimen. Third and more rarely, AKI may be the consequence of group A streptococcus-related AIN [8–10], as was the case in our patient. Distinguishing among these three entities is important because they have a different course, treatment and prognosis.

## Discussion

In this report, we describe a 3-year-old girl with acute haemorrhagic interstitial nephritis associated with a group A streptococcal infection. AIN is an immune-mediated disease of the renal interstitium and is responsible for 3–7 % of AKI cases in the paediatric patient age group [11, 12]. It is mainly caused by drugs, infections and systemic auto-immune diseases (Table 1). The clinical manifestations of AIN are rather non-specific and include fever, vomiting, polyuria or oliguria, abdominal or loin pain and, especially in drug-induced AIN, skin rash and arthralgia. Patients with AIN are usually normotensive. AIN is biochemically characterised by a varying degree of AKI, (tubular) proteinuria, haematuria, leukocyturia, hypostenuria, tubular dysfunction, ranging from isolated glucosuria to Fanconi syndrome, (non-)haemolytic anaemia and eosinophilia/eosinophiluria. This latter finding is commonly associated with an allergic reaction underlying drug-induced AIN. Renal ultrasound may show normal-sized or enlarged kidneys with hyperechogenicity. The histological hallmarks of AIN are interstitial inflammation with a predominant mononuclear cell and eosinophil infiltrate, interstitial oedema with or without fibrosis, tubulitis, minimal or absent glomerular alterations and normal renal vessels [9–12]. Given the non-specific nature of clinical signs and symptoms, kidney biopsy is required for a definitive diagnosis of AIN [10–12].

**Table 1:** Differential diagnosis of acute interstitial nephritis [9–13]

Aetiology (percentage of all causes)	Corresponding findings in our patient
Drugs (70 %) Mainly antibiotics, NSAIDs, PPIs, anticonvulsants, and diuretics	• No medication use • No eosinophilia in peripheral blood • No eosinophilic interstitial infiltration
Auto-immune diseases (20 %) Sjögren syndrome, SLE, sarcoidosis, MCTD	• ANA and anti-dsDNA antibodies negative • Normal ACE, C3, C4, and C3d • No complement, IgA, IgG, or IgM deposition in renal biopsy specimen • No uveitis, arthritis or typical skin lesions • No hilar lymphadenopathy on chest X-ray
Infection (4–10 %) Viral: hantavirus, CMV, EBV, hepatitis viruses, polyomavirus, HIV, HFV Bacteria: *Mycoplasma*, group A *Streptococcus*, *Legionella*, *Leptospira*, *Brucella*, diphtheria, *Mycobacterium*, *Salmonella*, *Yersinia*, lues (syphilis), *Rickettsia* Fungal: histoplasma Parasitic: toxoplasma, leishmania	• Negative multiplex PCR for stool viruses • Negative PCR for adeno- and BK-virus in blood • Negative serology for CMV, EBV, mycoplasma • Negative PCR for hantavirus in renal biopsy • Hantavirus IgM weakly positive, IgG negative • Positive blood culture for group A beta-hemolytic streptococcus (Streptococcus pyogenes)
TINU syndrome (2 %)	• No signs of uveitis on eye examination
IgG4-related tubulointerstitial nephritis	• Total serum IgG normal • IgG subclasses not determined
Idiopathic hypocomplementemic tubulointerstitial nephritis	• Normal C3 and C4
ANCA-associated vasculitis	• Negative ANCA

ACE, Angiotensin converting enzyme; ANA, anti-nuclear antibodies; ANCA, anti-neutrophil cytoplasmic antibodies; CMV, cytomegalovirus; dsDNA, double-stranded DNA; EBV, Epstein–Barr virus; HFV, haemorrhagic fever viruses; HIV, human immunodeficiency virus; Ig, immunoglobulin; MCTD, mixed connective tissue disease; NSAIDs, non-steroidal anti-inflammatory drugs; PPIs, proton pump inhibitors; SLE, systemic lupus erythematosus; TINU, tubulointerstitial nephritis and uveitis

Treatment mainly consists of removal of the offending agent and supportive care with dialysis if needed. The role of corticosteroids in the treatment of AIN is controversial [10–12], except for cases with underlying auto-immune disease, with small retrospective studies showing therapeutic benefit. However, evidence from randomised controlled trials is lacking. Nevertheless, many clinicians opt for steroid treatment when confronted with severe renal failure and use a regimen similar to the one used in our case, namely, methylprednisolone pulses on 3 consecutive days, followed by oral prednisone tapered over several weeks [12]. With appropriate management, AIN has an excellent prognosis in the majority of children, with complete convalescence of renal function and normalisation of the urinary sediment within several weeks to months [10–12]. Clinical and/or histopathological parameters consistently predicting renal outcome in AIN have not yet been established. Consequently, life-long follow-up is recommended for all AIN patients [10, 12].

Although the literature on AIN secondary to streptococcal infection is relatively scarce, several reports have documented this association, both in adults [8, 9] and children [10]. In fact, AIN was first described as a clinical entity in patients with scarlet fever [8, 12]. We feel confident that our patient represents a further case of group A streptococcus-associated AIN because she presented with scarlet fever and her blood culture was positive for *Streptococcus pyogenes*. Additional support for the causative role of streptococci in the genesis of AIN can usually not be derived from the histopathological (i.e. light microscopic) findings since clues for a specific aetiology are generally lacking in renal biopsy specimens [10]. With immunofluorescence staining, the renal interstitium can be assessed for deposition of complement and immunoglobulins. Immunofluorescence studies were negative in our patient. Chang et al. [8] and Dharmarajan et al. [9] also failed to detect significant deposition of IgA, IgG, IgM and complement in their adult patients with AIN and streptococcal infection. The most comprehensive case series of AIN in children to date is that of Ellis et al. who described 13 children with biopsy-proven AIN, seven of whom had clinical and/or laboratory signs of streptococcal infection [10]. Results of immunofluorescence staining are mentioned in their report, although not linked to the underlying aetiologies. However, these authors did find interstitial deposits of immunoglobulins (IgG) and complement (C4) in merely one of the 13 cases. Hence, the majority of children with AIN due to streptococcal infection showed no staining for either immunoglobulins or complement factors. The only direct evidence of a causal relationship between group A streptococcal infection and AIN has been provided by Chang et al. [8]. who performed immunohistochemical studies in an adult patient with streptococcal toxic shock syndrome and light microscopic evidence of AIN. The results of these studies demonstrated the presence of streptococcal pyrogenic exotoxin B in the tubulointerstitial compartment. Unfortunately, we were not able to apply this immunohistochemical technique to the kidney biopsy specimen of our patient.

In conclusion, this report serves to illustrate that AKI associated with streptococcal infection is not always due to APSGN or ATN, but that it can incidentally be caused by AIN. Moreover, our unique clinical teaching point is that renal interstitial haemorrhage can be a manifestation of group A streptococcal infection. The latter should therefore be added to the differential diagnosis of HIN.
